# Differential expression of the ubiquitin-editing enzyme A20 in gastric biopsies indicates the severity of disease

**DOI:** 10.1007/s00418-024-02345-2

**Published:** 2024-12-31

**Authors:** Stephan Schnizler, Michael Naumann, Michael Vieth

**Affiliations:** 1 Institute of Pathology, Klinikum Bayreuth, 95445 Bayreuth, Germany; 2 Institute of Experimental Internal Medicine, Otto-Von-Guericke-Universität Magdeburg, 39120 Magdeburg, Germany; 3 Friedrich-Alexander-Universität Erlangen-Nürnberg, 91054 Erlangen, Germany

**Keywords:** A20, Gastric adenocarcinoma, Gastric mucosa, *Helicobacter* B-type gastritis, NF-kB

## Abstract

A20, an ubiquitin-editing enzyme, plays a pivotal role in regulating cell signaling and immune responses. Dysregulated A20 expression has been associated with various pathological conditions, including inflammatory diseases and malignancies, where its expression levels often correlate with differing prognoses in solid tumors. This study aimed to explore the expression and cellular localization of A20 in both nonpathological and diseased human gastric tissues to gain deeper insights into its involvement in gastric pathologies. We analyzed paraffin-embedded gastric tissue samples from 326 patients. A20 expression was assessed using immunohistochemistry (IHC) with results categorized according to the Remmele and Stegner immunoreactive score (IRS). The study compared A20 expression across a spectrum of gastric pathologies, including *Helicobacter pylori* (HP) gastritis, autoimmune gastritis (A-gastritis), reactive gastropathy (C-gastritis), Ex-HP-gastritis, adenomas, and adenocarcinomas, with nonpathological gastric mucosa serving as a baseline. Our findings demonstrate a significant increase in A20 expression in HP-gastritis (*p* = 0.019), A-gastritis (*p* = 0.001), adenomas (*p* < 0.001), and adenocarcinomas (*p* < 0.001). Conversely, no significant differences in A20 expression were observed in C-gastritis or Ex-HP-gastritis cases.

**Supplementary Information:**

The online version contains supplementary material available at 10.1007/s00418-024-02345-2.

## Introduction

A20 is an ubiquitin-editing protein encoded by the A20 gene tumor necrosis factor alpha-induced protein 3 (*TNFAIP3*). It was initially identified as a primary tumor necrosis factor (TNF) responsive gene in human umbilical vein endothelial cells (Dixit et al. 1990). The zinc finger protein A20 regulates substrate proteins in a catalytic or noncatalytic manner. At the N-terminus, it contains an ovarian tumor (OTU) domain that exerts deubiquitinylase activity, and the C-terminal zinc finger (ZnF) domains ZnF4 and ZnF7 function as ubiquitin-binding domains (Martens and van Loo 2020).

A20 plays pivotal roles in regulating inflammation, innate immunity, and adaptive immunity (Priem et al. 2020; Schlüter et al. 2022). A20 exerts its effects through different mechanisms in response to various stimuli (Priem et al. 2020). For example A20 interferes with the components of the nuclear factor kappa-light-chain-enhancer of activated B cells (NF-κB) signalling cascade and terminates NF-κB activation, e.g., by interacting with the linear polyubiquitin chain of NF-κB essential modulator (NEMO), thereby preventing activation of the IκB kinases (IKKs) (Tokunaga et al. 2012). These processes prevent excessive NF-κB activation, thereby maintaining a balance in the immune response. The significance of A20 in homeodynamics is underscored by its associations with various autoimmune diseases, including Crohn’s disease, systemic lupus erythematosus, rheumatoid arthritis, and type 1 diabetes (Vereecke et al. 2011).

In addition to its role in the regulation of factors involved in immune responses, A20 enzymatically counteracts cullin3-mediated K63-linked ubiquitinylation of procaspase-8, suppressing caspase-8 activity and apoptotic cell death (Lim et al. 2017, 2018). Here, A20 could be a critical inhibitor of cell death in many cell types. Although its precise mechanisms of action are not yet fully understood, A20 has been identified as an apoptosis inhibitor in endothelial cells (Dixit et al. 1990), thymocytes, fibroblasts (Lee et al. 2000), pancreatic β-cells (Fukaya et al. 2016), hepatocytes (Catrysse et al. 2016), and intestinal epithelial cells (Vereecke et al. 2010). In addition to blocking TNF-induced, caspase-mediated apoptosis, A20 is also suggested to inhibit caspase-independent necroptosis (Onizawa et al. 2015).

While acting as a tumor suppressor in B-cell Lymphomas (Kato et al. 2009) owing to its ability to promote apoptosis and inhibit TNF-mediated cell death, its precise role in cancer remains unresolved. Recent studies imply that A20 can have both oncogenic and tumor-suppressive functions depending on the tissue context (Shi et al. 2021). For example, elevated A20 expression has been detected using similar immunohistochemical methods in breast cancer, where it functions as an oncogene (Sharif-Askari et al. 2021), and in melanoma (Ma et al. 2020). Conversely, in hepatocellular carcinomas, despite its increased expression, A20 shows antitumoral effects (Chen et al. 2015). Reduced A20 expression has been seen with immunohistochemistry in pancreatic cancer tissue (Wang et al. 2012). There is limited data on A20 expression in human gastric mucosal tissue. However, exploring A20 in various gastric pathologies is compelling because of its potential regulatory roles in inflammation and cancer. Persistent *H*. *pylori* infection in HP- gastritis (type B-gastritis) is a known risk factor for cancer development. Studying A20 in Ex-HP-gastritis helps to understand its role in post-eradication inflammation. Examining A20 in adenomas and adenocarcinomas could provide valuable insights into cancer progression, potentially leading to new diagnostic and therapeutic strategies.

Previous studies have shown that A20 contributes to the negative regulation of alternative NF-κB signaling in gastric epithelial cells infected by *H*. *pylori* (Lim et al. 2022). Moreover, A20 acts as an important negative regulator of caspase 8 (Jantaree et al. 2022) and suppresses apoptosis in gastric tumor cells. These findings suggest that studying A20 in gastric tissues may provide critical insights into gastric pathophysiology.

Given the diverse roles of A20 in inflammation and cancer, as well as its diagnostic and prognostic potential, this study aims to elucidate the expression of A20 protein in human gastric mucosa tissue from patients with reactive and inflammatory gastropathies, as well as patients with gastric neoplasia.

## Materials and methods

### Tissue samples and data acquisition

A retrospective series of gastric tissue samples from the archives of the Institute of Pathology in Bayreuth was used in this study. The tissues were originally fixed in 4% formalin and embedded in paraffin, according to established methods (Ramos-Vara 2011).

A total of 326 paraffin embedded tissue samples containing gastric mucosa without pathological change, C-gastritis, HP-gastritis, Ex-HP-gastritis, A-gastritis, adenomas, and adenocarcinomas were used.

All hematoxylin and eosin (HE) samples were analyzed in the pathology department independently by two pathologists. The samples were randomly selected and include tissue gained from routine clinical management for either diagnostic or therapeutic purposes from the years 2014–2016.

### Ethics statement

The study was approved by the ethics committee of the medical faculty of the Friedrich-Alexander University Erlangen (347_20 Bc).

### Immunohistochemistry

Paraffin embedded, 3 µm thick sections were prepared on a microtome (Leica Mikrosysteme Vertrieb GmbH, Wetzlar, Germany) and used for immunohistochemistry.

The expression intensity and cellular location were determined using immunohistochemistry. One section was previously HE-stained from our routine diagnostic workflow and another section was immunohistochemically prepared and examined for A20 protein expression.

Sections were deparaffinized and stained with the automated LEICA Bond III (Leica Mikrosysteme Vertrieb GmbH, Wetzlar, Germany) using a monoclonal A20 antibody in a 1:100 dilution after being pretreated with EDTA-buffer for 20 min. The manufacturer of the A20 antibody (sc-166692, Santa Cruz Biotechnology, Inc., Dallas, TX, USA) has provided proof of validation on the technical specifications. We used human appendix and lung tissue as positive tissue specificity controls, as suggested by the manufacturer. For a negative tissue control, we omitted the primary antibody to rule out non-specific staining (Fig. S1).

Whole slide scanning and image acquisition was performed with Hamamatsu Nanozoomer S360 scanner and software (Hamamatsu Photonics Deutschland GmbH, Herrsching, Germany), featuring a 20X (NA 0,75) objective lens at a scanning resolution of 0,23 µm/pixel.

### Evaluation of immunostaining

Immunhistochemically stained slides were evaluated independently by two pathologists. Image analysis was performed with NDP.view2 Image viewing software (Hamamatsu Photonics Deutschland GmbH, Herrsching, Germany).

We evaluated the immunoreactivity of all samples using the immunoreactive score (IRS) by Remmele and Stegner (Remmele and Stegner 1987), a semiquantitative method that combines staining intensity and the proportion of positively stained cells to produce a 12-point score. Staining intensity was assessed on a scale from 0 to 3 (0 = negative, 1 = weak, 2 = intermediate, 3 = strong), while the percentage of stained cells was measured on a scale from 0 to 4 (0 = none, 1 = less than 10%, 2 = 10–50%, 3 = 51–80%, 4 = more than 80%).

The IRS was then calculated by multiplying the intensity score by the percentage score, resulting in a range from 0 to 12. On the basis of the IRS, staining was categorized as negative (IRS 0–2), weak (IRS 3–4), moderate (IRS 6–8), or strong (IRS 9–12). This method allowed us to quantify and compare the level of immunoreactivity across different sample groups effectively.

Randomly selected specimens were again reviewed by an experienced pathologist for confirmation.

### Statistical analysis

All independent, non-parametric samples were analyzed for statistical significance using the Mann–Whitney-*U* test. The threshold for statistical significance was set as *p* < 0,05. In the boxplots, the boxed area corresponds to the 25th to 75th percentile, with the white line depicting the mean value. The whiskers demonstrate the maximal and minimal value obtained.

## Results and discussion

### Patients with A-gastritis and HP-gastritis exert increased A20 expression

**Fig. 1:**
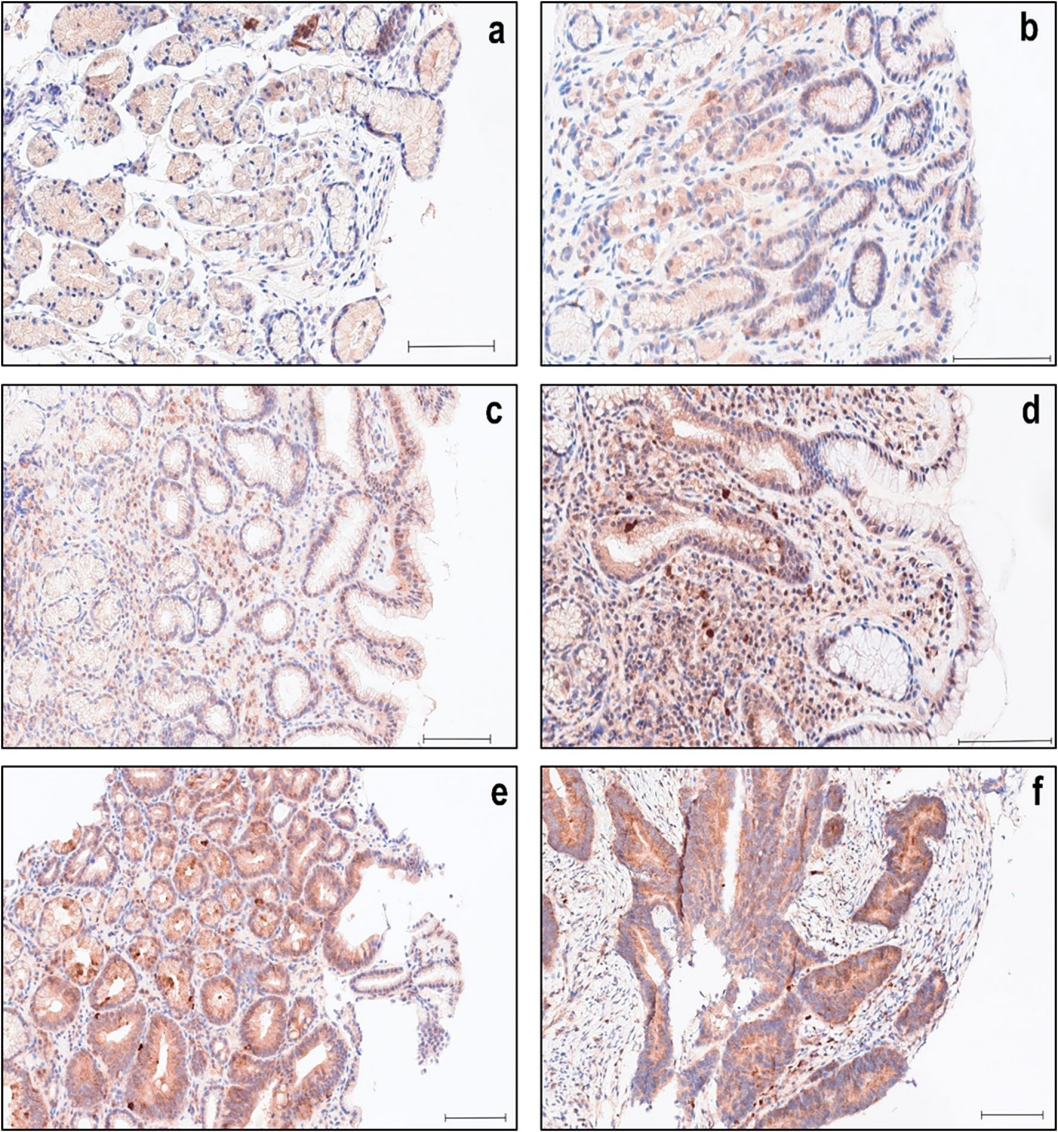
IHC of A20 in nonpathological- and pathological gastric antrum tissue. **a** Moderate, purely cytoplasmic staining intensity in nonpathological gastric epithelium, **b** as well as in C-gastritis and **c** Ex-HP-gastritis. **d** Slightly stronger cytoplasmic staining intensity in the strong background of inflammation of HP-gastritis. **e** Strong and diffuse cytoplasmic staining in adenoma and **f** adenocarcinoma. Scale bar: 100 µm

**Fig. 2:**
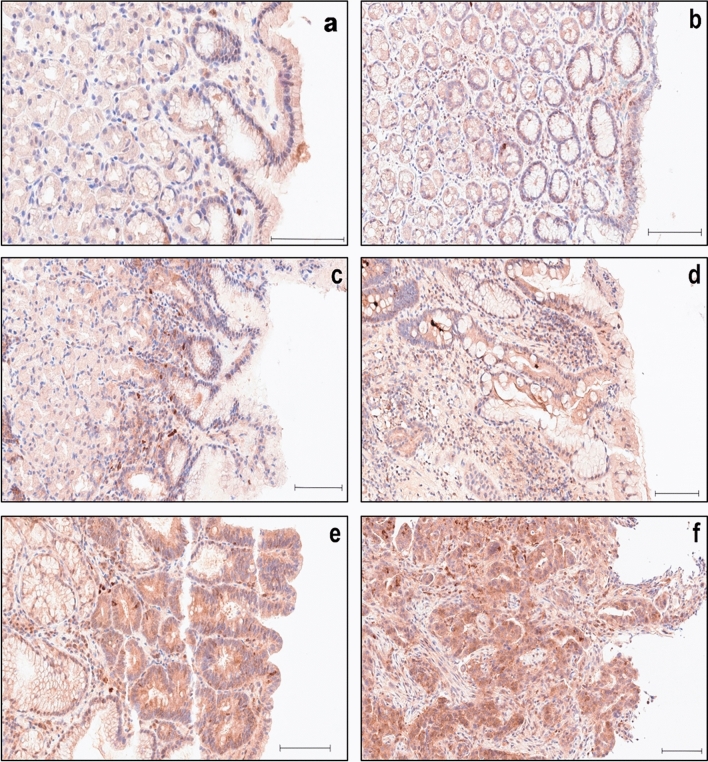
IHC of A20 in nonpathological- and pathological gastric corpus tissue. **a** Weak to moderate, purely cytoplasmic staining intensity in nonpathological gastric epithelium, **b** as well as in Ex-HP-gastritis. **c** Slightly stronger cytoplasmic staining intensity in the background of subepithelial inflammation of HP-gastritis **d** and in the intestinal metaplastic epithelium of A-gastritis. **e** Strong and diffuse cytoplasmic staining in adenoma and **f** adenocarcinoma. Scale bar: 100 µm

In nonpathological tissue of the gastric mucosa, A20 exhibited predominantly diffuse cytoplasmic staining of moderate intensity (Fig. 1a, Fig. 2a), with an IRS of 6–8 observed in 76,7% of cases (Table 1), correlating with the expected localization (Verstrepen et al. 2010). No nuclear staining was detected. There were no notable differences in staining intensity between the deeper glandular epithelium and the surface foveolar epithelium, nor between the antrum and the body of the stomach. In addition to the columnar cells of the gastric epithelium, weak A20 staining was also present in scattered lymphocytes and plasma cells of the lamina propria, and neuroendocrine cells (Fig. 1a, Fig. 2a).

**Table 1:** IHC staining intensity of A20 indicated by number of specimens and percentage in nonpathological and different pathological gastric tissues

Diagnosis	Number	Age (years)	Sex			Staining intensity	
			M	F	Weak	Moderate	Strong
Normal	43	29–62	21	22	8 (18,6%)	33 (76,7%)	2 (4,7%)
Antrum	20				4 (20%)	15 (75%)	1 (5%)
Corpus	23				4 (17,4%)	18 (78,3%)	1 (4,3%)
C-gastritis	48	15–84	22	26	10 (20,8%)	35 (72,9%)	3 (6,3%)
Antrum	48				10	35/3*	3
Corpus	0						
Ex-HP-gastritis	48	15–90	23	25	10 (20,8%)	38 (79,2%)	0
Antrum	28				8 (28,6%)/2*	20 (71,4%)/6*	0
Corpus	20				2 (10%)	18 (90%)/1*	0
HP-gastritis	49	13–90	25	25	5 (10,2%)	34 (69,4%)	10 (20,4%)
Antrum	28				3 (10,7%)	18 (64,3%)/6*	6 (21,4%)/1*
Corpus	21				1 (4,8%)	16 (76,2%)	4 (19%)
A-gastritis	52	27–85	14	38	1 (1,9%)	36 (69,2%)	15 (28,9%)
Antrum	0				1 (1,9%)	36 (69,2%)/22*	15 (28,9%)/12*
Corpus	52						
Adenoma	46	40–97	22	24	2 (4,3%)	17 (37%)	27 (58,7%)
Antrum	30				1 (3,3%)	10 (33,3%)	19 (63,3%)
Corpus	16				1 (6,3%)	7 (43,8%)	8 (50%)
Adenocarcinoma	40	42–92	28	12	2 (5%)	21 (52,5%)	17 (42,5%)
Antrum	21				1 (4,8%)	12 (57,1%)	8 (38,1%)
Corpus	19				1 (5,3%)	9 (47,4%)	9 (47,4%)

* cases with intestinal metaplasia

**Fig. 3:**
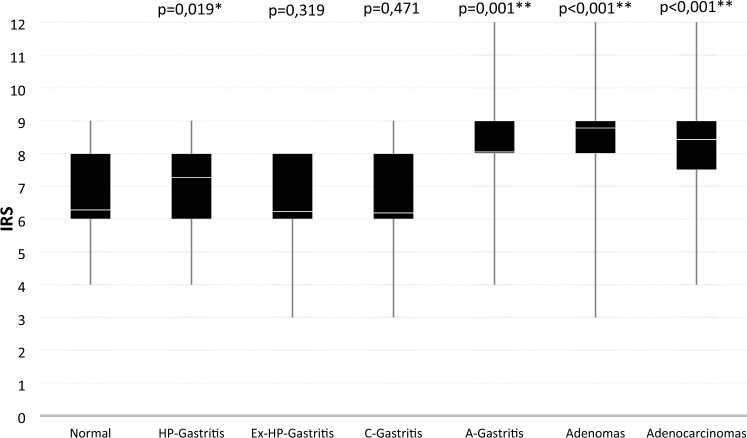
Box plot depicting the distribution of the IRS in different gastric pathologies. The boxes representing the interquartile range, with the white line in the box representing the mean value. The whiskers extend from the smallest to largest value. Asterisks marking the categories where the difference from the normal group is statistically significant (*) or very significant (**)

**Fig. 4:**
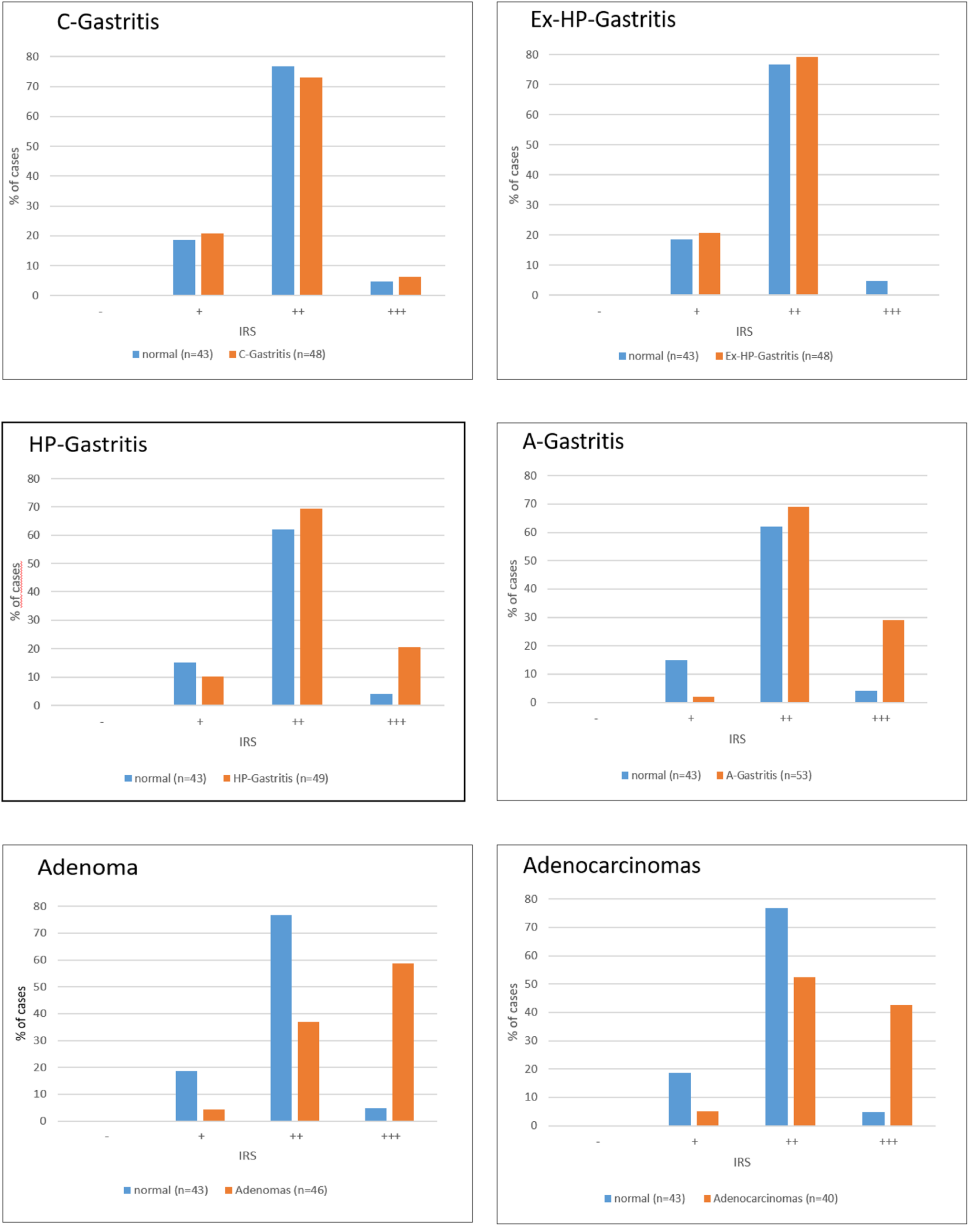
IRS of A20 in different gastric pathologies. Comparison of the percentage of cases across different gastric pathologies: C-gastritis, Ex-HP-gastritis, HP-gastritis, A-gastritis, adenoma, and adenocarcinomas. Each graph displays the distribution of normal (*n* = 43) cases versus the specific condition, categorized by immunoreactivity score (IRS) levels: negative (−), weak ( +), moderate (+ +), and strong (+ + +)

A similar expression profile was noted in C-gastritis (Fig. 1b) (solely diagnosed in the gastric antrum) and Ex-HP-gastritis (Fig. 1c, Fig. 2b), leading to no significant differences observed in C-gastritis (*p* = 0,471) and Ex-HP-gastritis (*p* = 0,319) (Fig. 3) and similar distributions in IRS scoring (Fig. 4).

In cases of HP-gastritis, we discovered an increase in staining intensity of epithelial cells in a subset of cases (Fig. 3) within the area of characteristic active subepithelial band-like inflammation (Fig. 1d, Fig. 2c), resulting in significant higher IRS in comparison to normal gastric mucosa (*p* = 0.019, Fig. 4). This finding can be explained by A20’s role as an early-response gene, de novo synthesized during inflammation. A20 is upregulated in inflammatory conditions through NF-κB activation, acting within a crucial negative feedback loop that regulates NF-κB signaling. In gastric epithelial cells, *H*. *pylori* infection stimulates both classical and alternative NF-κB pathways via adenosine diphosphate (ADP)-D-glycero-β-D-manno-heptose or ADP-L-glycero-β-D-manno-heptose (Lim et al. 2022). Herein, A20 acts as a key regulator by suppressing NF-κB activity and thereby impacts inflammatory immune responses (Lim et al. 2023; Maubach et al. 2022).

In nonneoplastic lesions, A20 staining was generally more intense in intestinal metaplasia, a condition in which the normal gastric mucosa is replaced by intestinal type epithelium replete with mucin producing goblet cells (Fig. 2d). Intestinal metaplasia was identified in a substantial number (Table 1) of the examined gastritis cases (A-gastritis: 65%, HP-gastritis: 14%, C-gastritis: 6%, Ex-HP-gastritis: 19%). Intestinal metaplasia commonly occurs as a response to chronic *H*. *pylori* infection, bile acid reflux, smoking, or high salt intake, and is considered a precancerous lesion associated with an elevated risk of gastric carcinoma (Nieuwenburg et al. 2021).

A notable increase in A20 expression was therefore observed in A-gastritis (*p* = 0.001, Fig. 3), diagnosed solely in the gastric corpus, where gastric intestinal metaplasia is a common finding and often extensive (Fig. 2d). Importantly, the nonmetaplastic foveolar epithelium in A-gastritis did not exhibit increased A20 expression.

In summary, A20 showed moderate diffuse cytoplasmic staining with consistent intensity across all regions in nonpathological gastric mucosa. Significant A20 increases were seen in A-gastritis and HP-gastritis, but not in C-gastritis or Ex-HP-gastritis. Owing to the lack of notable differences in staining intensity between the antrum and body of the stomach (Table 1), the data presented in Figs. 1–3 omit subdivision of cases for clarity purposes.

### Elevated A20 expression in gastric neoplastic lesions

A large proportion of adenomas demonstrated strong cytoplasmic staining (Fig. 1e, Fig. 2e), resulting in a significantly higher IRS (Fig. 4) compared with adjacent normal epithelium (*p* < 0,0001, Fig. 3). There was no nuclear staining observed in either adenomas or adenocarcinomas (Fig. 1e, f and Fig. 2e, f).

In intestinal type carcinomas, a significant increase in A20 expression was noted as well (*p* < 0,001, Fig. 1f, Fig. 2f, Fig. 3). Owing to difficulty in interpretation of cytoplasmic staining of signet ring cells, in which the cytoplasm is predominantly replaced by intracellular mucin and no reliable evaluation was possible, diffuse-type carcinomas were not included. Overall, both adenomas and intestinal-type carcinomas exhibited significantly higher A20 expression compared with adjacent normal tissue. No nuclear staining was observed in these neoplastic lesions.

Elevated A20 expression has been observed in several cancers, including breast cancer and melanoma. Herein, it acts as an oncogene by promoting cellular survival even under conditions that would typically induce apoptosis (Priem et al. 2019). Conversely, in hepatocellular carcinomas, A20 displays anti-tumoral effects despite its increased expression (Shi et al. 2021) while pancreatic cancer tissue exhibits reduced A20 levels (Wang et al. 2012).

Moreover, A20 plays a crucial role in enabling cancer cell resistance to DNA-damaging treatments. This resistance mechanism highlights the potential of targeting A20 in cancer treatment to improve therapeutic outcomes (Yang et al. 2018). Given our finding that A20 is also upregulated in gastric adenomas, this underscores the potential involvement of A20 in the development and progression of gastric cancer. Thus, A20 could be a potential therapeutic target. However, systemic inhibition of A20 could be problematic, as evidenced by knockout mice experiments, where the absence of A20 led to detrimental effects. A20 deficient mice experience severe inflammation and significant weight loss (cachexia), show an extreme sensitivity to inflammatory signals and typically die early. Without A20, cells are unable to properly terminate the NF-κB inflammatory response, making them more prone to cell death (Lee et al. 2000).

While targeting of A20 in cancer therapy might be promising, it must be approached with caution to avoid adverse systemic consequences. Targeting A20 would not only affect the tumor but also the surrounding tissue. Therefore, to identify adverse effects, studies using cocultures with organoids/mucosoids and additional cell types could prove valuable to gain relevant insights to mitigate adverse effects (Jantaree et al. 2021; Maubach and Naumann 2024).

## Supplementary Information

Below is the link to the electronic supplementary material.

Supplementary file1 (DOCX 279 KB)

## Data Availability

All data generated or analyzed during this study are included in this published article.
