# Correction: Differential expression of the ubiquitin-editing enzyme A20 in gastric biopsies indicates the severity of disease

**DOI:** 10.1007/s00418-025-02369-2

**Published:** 2025-04-08

**Authors:** Stephan Schnizler, Michael Naumann, Michael Vieth

**Affiliations:** 1 Institute of Pathology, Klinikum Bayreuth, 95445 Bayreuth, Germany; 2 Institute of Experimental Internal Medicine, Otto-Von-Guericke-Universität Magdeburg, 39120 Magdeburg, Germany; 3 Friedrich-Alexander-Universität Erlangen-Nürnberg, 91054 Erlangen, Germany

**Correction: Histochemistry and Cell Biology (2024) 163:22** 10.1007/s00418-024-02345-2

The article “Differential expression of the ubiquitin-editing enzyme A20 in gastric biopsies indicates the severity of disease “, written by Stephan Schnizler, Michael Naumann and Michael Vieth, was originally published under exclusive license to © The Author(s), under exclusive licence to Springer-Verlag GmbH Germany, part of Springer Nature 2024. As a result of the subsequent decision to publish the article under the open access model, the article’s copyright notice was changed on 27th February to “© The Author(s)” 2024 and the article is now distributed under a Creative Commons Attribution 4.0 International License, which permits use, sharing, adaptation, distribution and reproduction in any medium or format, as long as you give appropriate credit to the original author(s) and the source, provide a link to the Creative Commons licence, and indicate if changes were made [52].

The images or other third party material in this article are included in the article’s Creative Commons licence, unless indicated otherwise in a credit line to the material. If material is not included in the article’s Creative Commons licence and your intended use is not permitted by statutory regulation or exceeds the permitted use, you will need to obtain permission directly from the copyright holder. To view a copy of this licence, visit http://creativecommons.org/licenses/by/4.0.

