# Supplementary material for: Differential expression of the ubiquitin-editing enzyme A20 in gastric biopsies indicates the severity of disease
Source: Histochem Cell Biol. 2024 Dec 31;163(1):22. doi: 10.1007/s00418-024-02345-2 (PMC11978676; doi:10.1007/s00418-024-02345-2)

Supplementary material

Differential expression of the ubiquitin-editing enzyme A20 in gastric biopsies indicates the severity of disease

Journal

Histochemistry and Cell Biology

Stephan Schnizler^1,3^ , Michael Naumann^1,2*#^, Michael Vieth^1,3*^

^1^ Institute of Pathology, Klinikum Bayreuth, 95445 Bayreuth, Germany

^2^ Institute of Experimental Internal Medicine, Otto-von-Guericke-Universität Magdeburg, 39120 Magdeburg, Germany

^3^ Friedrich-Alexander-Universität Erlangen-Nürnberg, 91054 Erlangen, Germany

*Both authors contributed equally

^#^corresponding author:

Michael Naumann


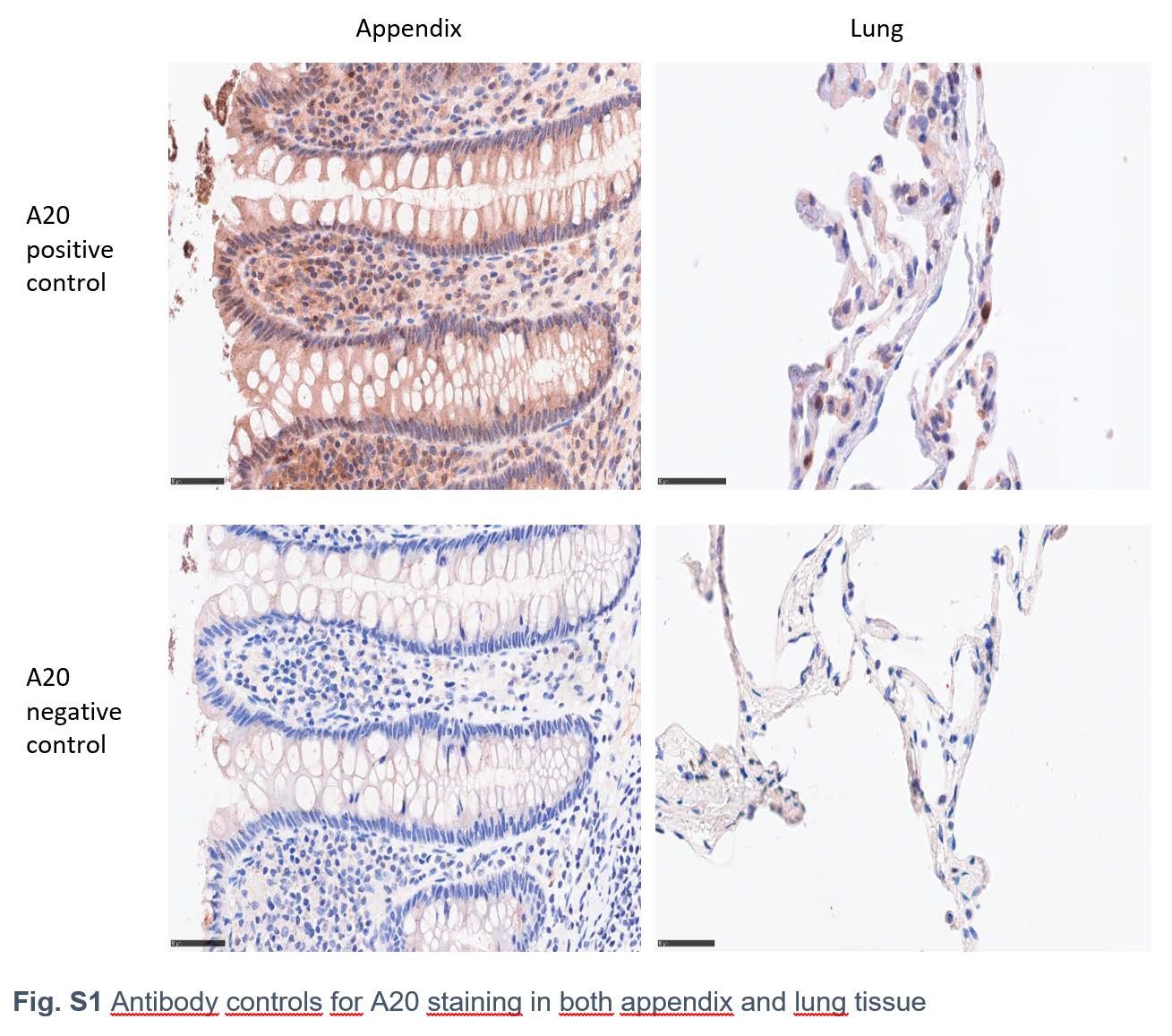

Supplement: Supplementary file 1 — Supplementary file1 (DOCX 279 KB) [file 418_2024_2345_MOESM1_ESM.docx]
